# The efficacy and therapy management of nab-paclitaxel in the real-world setting for patients with advanced breast cancer – the SERAPHINA study

**DOI:** 10.1007/s00432-025-06246-2

**Published:** 2025-06-19

**Authors:** Andreas Schneeweiss, Peter A. Fasching, Marc Thill, Marion van Mackelenbergh, Frederik Marme, Hans Tesch, Tanja N. Fehm, Tjoung-Won Park-Simon, Lothar Häberle, Sabrina Uhrig, Oliver Tome, Thomas Spall, Anna-Katharin Theuser, Matthias Ruebner, Erik Belleville, Diethelm Wallwiener, Sara Y. Brucker, Andreas D. Hartkopf

**Affiliations:** 1 National Center for Tumor Diseases (NCT), University Hospital and German Cancer Research Center, Heidelberg, Germany; 2 Department of Gynecology and Obstetrics, Universitätsklinikum Erlangen, Friedrich- Alexander-Universität Erlangen-Nürnberg (FAU), Erlangen, Germany; 3 Comprehensive Cancer Center Erlangen-EMN (CCC ER-EMN), Erlangen, Germany; 4 Department of Gynecology and Gynecological Oncology, AGAPLESION MARKUS KRANKENHAUS, Frankfurt, Germany; 5 Department of Gynecology and Obstetrics, University Hospital Schleswig-Holstein, Campus Kiel, Kiel, Germany; 6 Department of Gynecology and Obstetrics, University Hospital Mannheim, Mannheim, Germany; 7 Oncology Practice, Bethanien Hospital, Frankfurt am Main, Germany; 8 Department of Gynecology and Obstetrics, University Hospital Düsseldorf, Düsseldorf, Germany; 9 Center for Integrated Oncology (CIO Aachen, Bonn, Cologne, Düsseldorf), Düsseldorf, Germany; 10 Department of Gynecology and Obstetrics, Hannover Medical School, Hannover, Germany; 11 Biostatistics Unit, Department of Gynecology and Obstetrics, Universitätsklinikum Erlangen, Friedrich-Alexander-Universität Erlangen-Nürnberg (FAU), Erlangen, Germany; 12 Department of Gynecology and Obstetrics, St. Vincentius-Kliniken gAG , Karlsruhe, Germany; 13 ClinSol GmbH & Co KG, Würzburg, Germany; 14 Institut für Frauengesundheit GmbH, Erlangen, Germany; 15 Department of Gynecology and Obstetrics, University Hospital Tübingen, Tübingen, Germany; 16 Department of Gynecology and Obstetrics, Comprehensive Cancer Center Erlangen-EMN, Universitätsklinikum Erlangen, Friedrich-Alexander-Universität Erlangen-Nürnberg, Universitaetsstrasse 21–23, 91054 Erlangen, Germany

**Keywords:** Advanced breast cancer, Nab-paclitaxel, Chemotherapy, Real-world, Non-interventional study

## Abstract

**Background:**

Chemotherapies are still widely used in advanced breast cancer, specifically in patients with HER2-negative disease. This non-interventional real-world study assessed the utilization of nab-paclitaxel in a broad patient population with advanced breast cancer.

**Methods:**

SERAPHINA (NCT02642406) was a single arm, non-interventional study performed in Germany. Patients were eligible if they were treated with nab-paclitaxel according to the Summary of Product Characteristics (SmPC) as indicated by their physician. Progression-free survival (PFS), overall survival (OS), safety and quality of life were evaluated. Additionally, efficacy was assessed in patient subgroups based on age, metastasis pattern, performance status and therapy line.

**Results:**

A total of 432 patients were treated with nab-paclitaxel. The majority of patients had HER2-negative disease (94.2%). Furthermore, 30.1% of patients were treated in the first line and 48.3% in the third or later therapy line. Median PFS was 6.0 months (95% CI: 5.6–6.9) and median OS was 15.3 months (95% CI: 12.5–17.5). Although no clear predictors of PFS and OS in multivariable Cox models could be identified, patients with brain metastases had the shortest PFS (3.0 months; 95% CI: 2.2–4.3). Adverse events occurred in 83.1% of patients, with high-grade adverse events being rare (< 8% of patients). Quality of life did not change under therapy.

**Conclusion:**

Nab-paclitaxel is used in patients with advanced breast cancer. In this real-world, non-interventional study, prognosis was favorable and safety profile manageable, which is comparable to previous clinical trials and real-world studies.

**Supplementary Information:**

The online version contains supplementary material available at 10.1007/s00432-025-06246-2.

## Purpose

In recent years, there have been major improvements in the treatment of both human epidermal growth factor receptor 2 (HER2)-negative molecular subtypes of breast cancer (i.e., triple negative breast cancer (TNBC) and hormone receptor-positive, HER2-negative (HRpos/HER2neg) breast cancer). In patients with TNBC, antibody-drug conjugates sacituzumab govitecan (Bardia et al. 2021) and trastuzumab deruxtecan (for HER2 low expression tumors) (Rugo et al. 2023) have been introduced. Additionally, the immune checkpoint inhibitors atezolizumab (in combination with nab-paclitaxel) (Schmid et al. 2018) and pembrolizumab (in combination with paclitaxel, nab-paclitaxel or carboplatin/gemcitabine) (Cortes et al. 2022) have also been approved for the first line treatment of patients with programmed death-ligand 1 (PD-L1)-positive, advanced TNBC. In patients with HRpos/HER2neg breast cancer, the introduction of cyclin-dependent kinase 4/6 (CDK4/6) inhibitors (Hortobagyi et al. 2016, 2022; Slamon et al. 2019, 2020; Slamon et al. 2018; Im et al. 2019; Tripathy et al. 2018; Sledge et al. 2017; Goetz et al. 2017; Finn et al. 2016; Turner et al. 2015; Nabieva and Fasching 2023; Engler et al. 2022; Schneeweiss et al. 2020), and as well trastuzumab deruxtecan (for HER2 low expressing tumors) (Modi et al. 2022) and sacituzumab govitecan (Rugo et al. 2023) have changed the treatment landscape in the advanced setting. Nevertheless, chemotherapy remains the only therapeutic option after exhaustion of endocrine-based treatments, immunotherapy, antibody-drug conjugate therapy, and poly (ADP-ribose) polymerase (PARP) inhibitor therapy, and is commonly used in the second (up to 47%) and third (up to 66%) lines of therapy in patients with TNBC or HRpos/HER2neg disease (Braun et al. 2022).

Therefore, classical chemotherapy is still one of the most frequently used treatment options in patients with TNBC as well as in patients with HRpos/HER2neg breast cancer. International guidelines recommend the use of single-agent chemotherapy regimens (Al Sukhun et al. 2024). Nevertheless, the choice of chemotherapy remains diverse and depends on prior chemotherapies, side effects and other factors. Since many patients with TNBC already receive taxane/anthracycline-based treatment regimens in the adjuvant setting (Ditsch et al. 2022) and *de novo* disease is uncommon in TNBC (Muller et al. 2022), the majority of patients with advanced TNBC have already been treated with these chemotherapies. Therefore, treatments such as platinum-based chemotherapies, bevacizumab-based regimens, eribulin-, gemcitabine- and vinorelbine-based regimens are often used in the metastatic setting when a chemotherapy is employed (Braun et al. 2022). In patients with advanced HRpos/HER2neg disease, taxane-based regimens are most frequently used, followed by combinations with anthracyclines, capecitabine, eribulin and other chemotherapies (Braun et al. 2022). Notably, many clinical trials use physicians’ choice chemotherapy in the comparator arm. For example, the OlympiaD, EMBRACA, Keynote-355, ASCENT, and Destiny-B04 trials were all studies, in which the investigational treatment was compared to a chemotherapy of physicians’ choice.

All of the above-mentioned circumstances require detailed knowledge of the current use of chemotherapies. Additionally, information on efficacy and side effects could help to plan treatment sequences that optimize efficacy and minimize side effects. For patients with advanced HER2-negative disease, nab-paclitaxel may be a good option. Real-world studies can help investigate the efficacy and side effect profile of nab-paclitaxel in different patient populations, which is currently still under-researched. Therefore, the aim of this study was to evaluate the patterns of use, efficacy and toxicity of nab-paclitaxel in patients with advanced breast cancer.

## Methods

### Study design

SERAPHINA (Safety, Efficacy And Patient Reported Outcomes of Advanced Breast Cancer Patients: Therapy Management With nab-Paclitaxel in Daily Routine; NCT02642406) was a single arm, non-interventional study that recruited across 76 study sites in Germany. The study was conducted in accordance with the Declaration of Helsinki and the Guidelines of the International Conference on Harmonization Good Clinical Practice (GCP). Approval of all respective ethics committees were obtained (initial approval obtained from the Ethical Committee of the Medical Faculty of the Friedrich-Alexander-Universität Erlangen-Nürnberg, Erlangen, Germany: date August 12 2015, approval number 193_15B) and all patients provided written informed consent. Patients were eligible for inclusion if they were being treated with nab-paclitaxel monotherapy according to the Summary of Product Characteristics (SmPC) as indicated by their physician. Notably, nab-paclitaxel therapy could not have been initiated more than 3 weeks prior to study entry, and patients had to have proven metastatic or locally advanced breast cancer. Patients were not allowed to participate in any other interventional study.

### Patient population

Between December 2015 and September 2018, 454 patients were registered in the study. Five patients did not meet all inclusion/exclusion criteria and were excluded as screening failures. A further 17 patients never started nab-paclitaxel therapy and were also excluded. Ultimately, the population for which safety was reported comprised 432 patients. For efficacy analyses, 10 patients who started the nab-paclitaxel more than 21 days before study entry and 2 patients, who had no follow-up information available were additionally excluded, resulting in *N* = 420 patients for efficacy analyses. A flow chart of the patient selection is shown in Supplementary Fig. 1.

### Data acquisition

Medical history, performance status, concomitant medication, and quality of life as assessed by FACT-B questionnaires were captured at study entry. This information, as well as the disease status, were assessed every three months until disease progression. Treatment was given according to the local standard. The recommended dose of nab-paclitaxel was 260 mg/m^2^, administered intravenously over 30 min every 3 weeks. However, other treatment schedules were documented as part of the SERAPHINA study as well. Patients were followed up every three months until disease progression, death, or withdrawal of consent. The end of study was defined to be either death, withdrawal of consent or a maximum observation time of 36 months. Disease status, including disease progression, was assessed at least every three months according to the local guidelines. The database cut for this analysis was September 20, 2022.

HER2 status and estrogen receptor (ER) and progesterone receptor (PR) expression were assessed according to the local practice standards. No central review was performed. ER and PR status were considered positive if ≥ 1% of the tumor cells were stained positively by immunohistochemistry. A positive HER2 status required an immunohistochemistry score of 3 or more or a positive fluorescence in situ hybridization/competitive in situ hybridization.

Adverse events, including the severity grading, were coded according to the National Cancer Institute Common Terminology Criteria for Adverse Events (version 4.03).

### Statistical considerations

The primary objective of this study was to describe progression-free survival (PFS) under real-life conditions. Secondary objectives included the evaluation of overall survival (OS), factors that influence PFS and OS, safety and quality of life.

PFS was defined from the date of start of therapy to the earliest date of disease progression (distant-metastasis, local recurrence, or death from any cause) or the last date known to be progression-free. It was left-truncated for time to enter the study, if the entry was after start of therapy. OS was defined in a similar fashion.

Survival rates with 95% confidence intervals (CIs) and median survival time were estimated using the Kaplan-Meier product limit method. The 95% CI of median survival time was computed using the method of Brookmeyer and Crowley (Brookmeyer and Crowley 1982).

For each outcome (PFS, OS), a multivariable Cox regression model was fitted with the following predictors: age at diagnosis (< 55 years, 55–65 years, > 65 years) body mass index (BMI, in kg/m2; < 20. 20–25, 25–30, > 30), grading (G1/G2; G3), HR status (HRpos; HRneg), HER2 status (HER2pos; HER2neg), nodal status (pN0, pN+), metastases pattern (brain; visceral; bone; other), therapy line (first, second, third or higher). Hazard ratios with 95% CI were calculated for each predictor using this Cox regression model. Moreover, the hazard ratio for non-triple-negative versus triple-negative patients was calculated by combining the regression coefficients accordingly.

Calculations were carried out using the R system for statistical computing (v4.2.1; R Core Team 2022)

## Results

### Patients

From December 2015 to September 2018, 454 patients were registered into the study, of whom 432 received nab-paclitaxel (safety population) and 420 patients had valid follow-up information available for efficacy analyses (efficacy population). A study flow chart is shown in Supplementary Fig. 1. Patients average age was 60.0 (± 11.6) years old. The majority of patients was HER2neg (94.2%) and HRpos (79.6%). Nab-paclitaxel treatment was started as a first-line therapy in 30.1% of patients, as a second-line therapy in 21.6% of patients, or as a third- or later-line therapy in 48.3% of patients. A summary of all patient characteristics is shown in Table 1.

### Therapy

A total of 432 patients started nab-paclitaxel therapy. The initial planned schedule for nab-paclitaxel therapy varied among patients. A weekly schedule was planned for most patients (*N* = 167; 38.8%), while a three-week schedule was planned for 105 patients (24.4%), and a four-week schedule (three weeks of treatment, followed by one week of rest) was planned for 149 patients (34.7%). Dose reductions were necessary in 36.6% of all patients. The mean duration of treatment was 20.9 (± 45.5) weeks and the mean relative dose intensity was 0.91% (± 0.44%). A summary of treatment characteristics is given in Supplementary Table 2.

### Efficacy

**Fig. 1:**
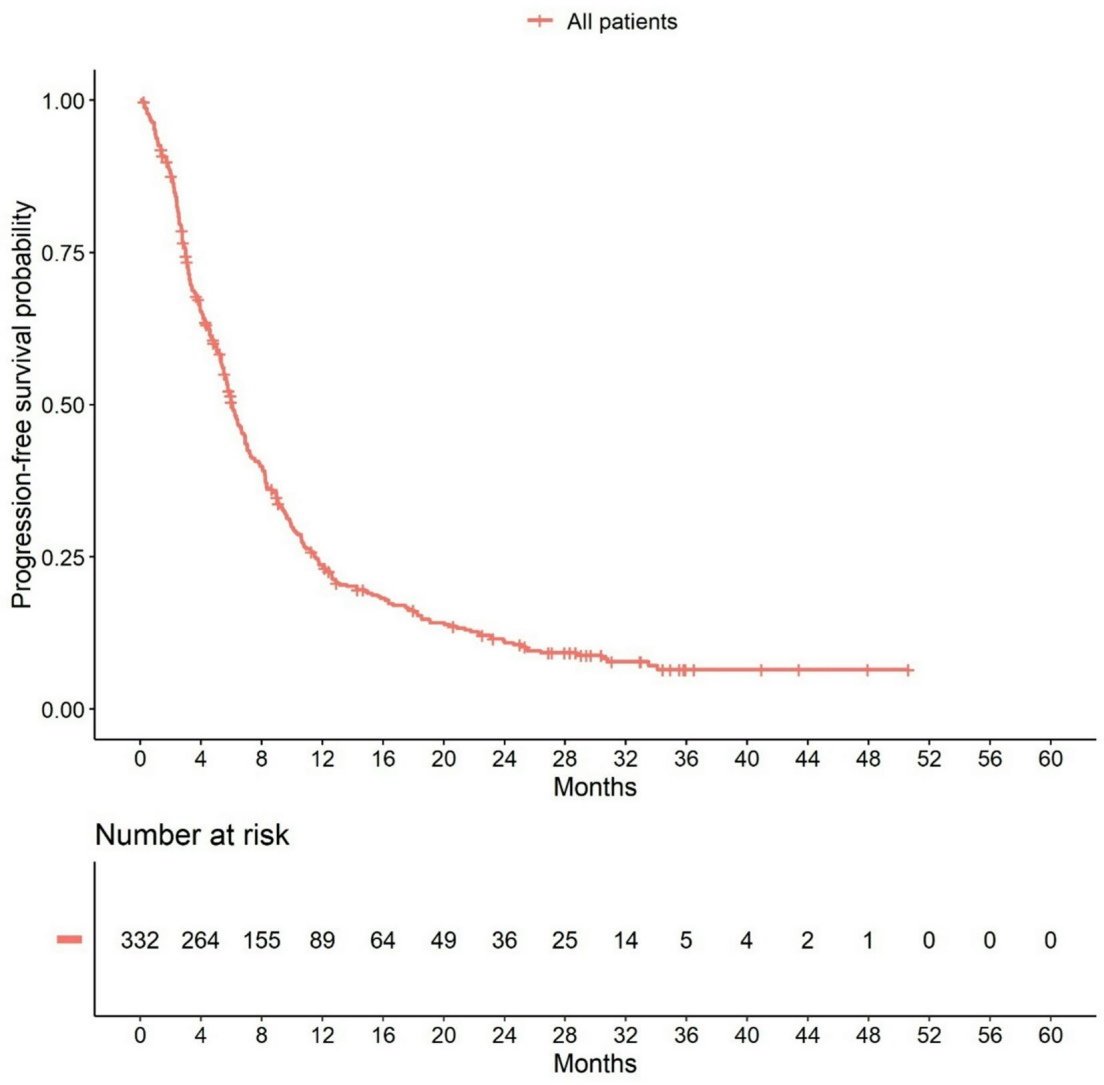
Progression-free survival in the efficacy population (*N* = 420)

**Fig. 2:**
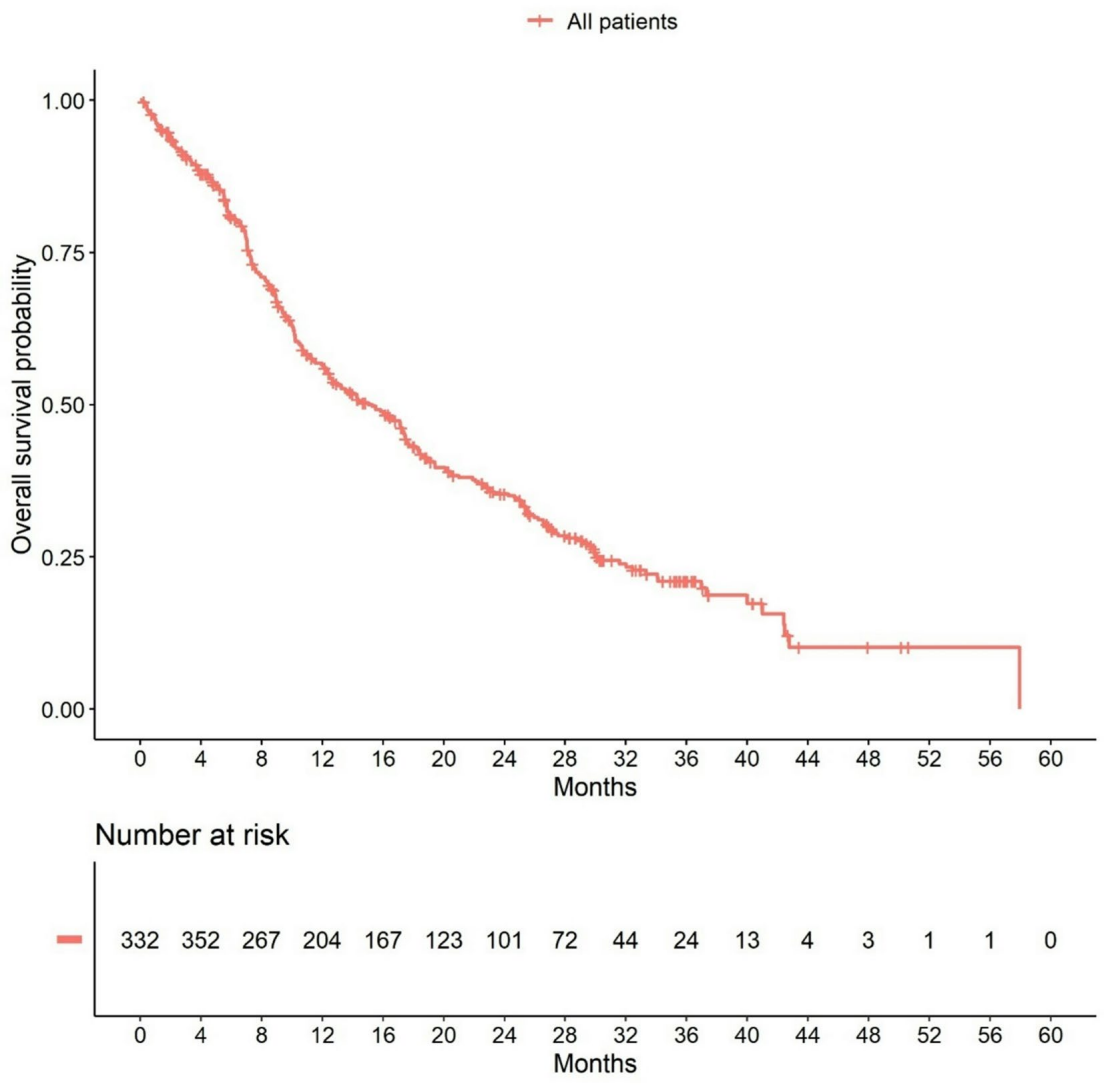
Overall survival in the efficacy population (*N* = 420)

The median observation time was 5.7 months for PFS and 11.2 months for OS. During this observation time, 359 disease progressions and 279 deaths occurred. Median PFS time was 6.0 months (95% CI: 5.6–6.9) and median OS time was 15.3 months (95% CI: 12.5–17.5). Survival rates are shown in Supplementary Tables 3 and Kaplan Meier Curves are shown in Figs. 1 and 2. Hazard ratios for predictors of PFS and OS are shown in Table 2. The hazard ratios for non-triple-negative versus triple-negative patients were 0.94 (95% CI: 0.52–1.70) for PFS and 0.87 (95% CI: 0.47–1.59) for OS. All median PFS and OS times and survival rates are shown in Supplementary Tables 4 and Supplementary Table 5. Median PFS and OS times were different for patients in different therapy lines and for patients with different metastases pattern. Patients in the first line had the longest median PFS (9.8 months (95% CI: 7.9–12.6)), while patients in the third or later therapy line had a median PFS time of 5.3 months (95% CI: 3.9-6.0; Supplementary Table 4). Similarly, therapy line was also associated with OS (first-line patients: median OS 25.7 months (95% CI: 20.5–37.0); third or later therapy line patients: median OS 11.4 months (95% CI: 9.7–14.2): Supplementary Table 5). Patients with brain metastases had the shortest median PFS time (3.0 months (95% CI: 2.2–4.3) vs. 6.0 month (95% CI: 5.5-7.0) for visceral metastases and 8.2 months (95% CI: 4.7–20.9) for bone metastases (Supplementary Table 4)), as well as the shortest OS time (7.6 months (95% CI: 5.8–13.2) vs. 13.1 months (95% CI: 10.9–17.1) for visceral metastases and 26.2 months (95% CI: 14.3-NA) for bone metastases; Supplementary Table 5). However, with the exception of metastasis pattern for OS, no variables that strongly influenced PFS or OS could be identified in the Cox regression model (Table 2). Kaplan-Meier curves for PFS and OS according to age (Supplementary Fig. 2), metastasis pattern (Supplementary Fig. 3), performance status (Karnofsky-Index; Supplementary Fig. 4) and therapy line (Supplementary Fig. 5) are presented in Supplementary Materials.

### Safety

Within the safety population (*N* = 432), 359 patients (83.1%) were affected by adverse events, irrespective of toxicity grade. All adverse events with a frequency of 3% or higher are shown in Table 3. The most common adverse event was peripheral neuropathy (*N* = 192; 44.4%), followed by the lack of wellbeing (malaise; *N* = 130; 30.1%), alopecia (*N* = 75; 17.4%), and nausea (*N* = 71; 16.4%). The most common high-grade adverse events (grade 3/4) were neutropenia (*N* = 33; 7.6%), peripheral sensory neuropathy (*N* = 19; 4.4%) and a decreased white blood cell count (*N* = 15; 3.5%).

### Quality of life

The results of the FACT-B questionnaire at baseline, month 3 and month 6 are shown in Table 4. At baseline, data was available of approximately 43–45% of patients, at month 3 from approximately 32–34% of patients and at month 6 of approximately 17–18% of patients. The trial outcome index (FACT-B TOI) was maintained between the assessment timepoints at baseline, at 3 months and at 6 months (60.8, 57.7 and 60.4 respectively). All other assessment parameters of the FACT-B questionnaire were also maintained over the time (Table 4).

## Discussion

In this non-interventional real-world study, we evaluated the use of nab-paclitaxel in a broad patient population. Nab-paclitaxel was commonly administered as an early-line therapy in breast cancer patients with HER2-negative disease. In addition, nab-paclitaxel therapy was associated with a favorable prognosis and a manageable safety profile. With the exception of metastasis pattern for OS, no variables were identified in the Cox regression analysis that influenced PFS or OS, although some differences in PFS and OS times for the metastasis pattern and therapy lines were observed.

Results from our study are comparable to data from previous clinical trials and other real-world analyses. The first international trial to demonstrate the superiority of nab-paclitaxel over paclitaxel (using a three-week treatment schedule) showed a median PFS time of 3.3 months in the nab-paclitaxel arm (Gradishar et al. 2005). Most of these patients (58%) were treated in advanced therapy lines, having previously received treatment(s) in the advanced therapy setting (Gradishar et al. 2005). In another international study conducted in the first-line setting (Gradishar et al. 2009), patients were treated with a weekly treatment schedule. Here, median PFS time was 12.9 months (Gradishar et al. 2009). In addition, a Korean real-world analysis of 102 patients reported a median PFS time of 4.0 months, with most patients having received more than three chemotherapies in the metastatic setting (Kim et al. 2022). Another real-world analysis from Greece with 150 patients, of whom the majority received at least 2 or more prior therapy lines for metastatic breast cancer, described a median PFS of 7.1 months for HRpos breast cancer and 5.0 months for TNBC (Koumarianou et al. 2020). In a retrospective cohort study with 664 patients that was performed in the USA, patients who received nab-paclitaxel monotherapy had a median time to the next therapy or death of 6.7 months in the first line, 5.8 months in the second line and 5.3 months in the third of later line (Liang et al. 2015). Our non-interventional study showed a median PFS time of 9.8 months (95% CI: 7.9–12.6) in the first-line setting and 5.3 months (95% CI: 3.9-6.0) in patients in the third- or later-line setting. In subgroup analyses, metastasis pattern, performance status (Karnofsky) and therapy line were of particular interest. Although not statistically significant in the multivariable Cox model, patients in earlier therapy lines had a better outcome than those in later lines. Furthermore, patients with brain metastases or a lower performance status had a less favorable prognosis. This is in line with many studies, which have been published in real-world studies (Laakmann et al. 2020; Witzel et al. 2018).

In the large randomized nab-paclitaxel trials (Gradishar et al. 2005, 2009), severe neutropenia (grade 3/4) was reported in 25–44% of patients. This is significantly higher than the 7.6% reported in this real-world study. However, febrile neutropenia that would have resulted in a serious medical condition was not reported. Therefore, the higher incidence in the clinical trials may result from more frequent blood testing for neutropenia. Peripheral sensory neuropathy was also more commonly reported in the clinical trials. Grade 3/4 neuropathy was seen in 8–17% of patients (Gradishar et al. 2005, 2009). In our study, this adverse event was observed in 4.4% of patients, which may be due to underreporting. Other real-world analyses have also reported lower numbers of severe febrile neutropenia, ranging between 0.7 and 5% (Bernardo et al. 2017; Koumarianou et al. 2020; Marschner et al. 2018).

While nab-paclitaxel remains one of several chemotherapy options for patients with HRpos/HER2neg disease when endocrine treatments have been exhausted, the introduction of immune checkpoint inhibitors has changed the therapy landscape of patients with advanced TNBC. Atezolizumab and pembrolizumab have been approved for the treatment of PD-L1 positive breast cancer (Cortes et al. 2022; Schmid et al. 2018). In both the Impassion130 and Keynote-355 trials, immune checkpoint inhibitor was used as the combination partner for chemotherapy. After the negative results of the Impassion131 study (Miles et al.^ 2021^), in which solvent paclitaxel was used as a combination partner, it was discussed whether the immunological properties of the combination partner (solvent paclitaxel vs. nab-paclitaxel) might be the reason for the negative results. However, in the Keynote-355 study, both chemotherapies were allowed and there was no difference in efficacy when using either nab-paclitaxel or solvent paclitaxel. Also in the neoadjuvant setting in which pembrolizumab is approved in combination with a platinum/anthracycline/paclitaxel-containing chemotherapy (Schmid et al. 2022; Schmid et al. 2020), there is data that supports the efficacy in combination with nab-paclitaxel (Fasching et al. 2023). Taken together, nab-paclitaxel might be a favorable combination partner for immune checkpoint inhibitors in the advanced therapy setting. Whether nab-paclitaxel or more advanced antibody-drug conjugates such as sacituzumab govitecan are the better choice for HER2-negative metastatic breast cancer remains unclear, although the ongoing ASCENT-03 and ASCENT-04 trials may provide more clarity.

Our study has several strengths and limitations. With 432 patients treated with nab-paclitaxel, our study is one of the larger real-world studies providing survival estimates. The favorable prognosis in our study is noteworthy because it is higher than previously reported in both clinical trials and real-world studies. A systematic comparison of the treatment effects in 21 randomized controlled trials and the corresponding effects in real-world datasets could show that real-world treatment benefits were 16% lower than those reported in randomized controlled trials with surrogate endpoints such as PFS (Lakdawalla et al. 2017). Therefore, our study may be biased toward a population with a more favorable prognosis. However, we performed a comprehensive analysis of patient subgroups to better assess the prognosis across the variability of the patient population. Further studies with more samples that examine additional predictors (e.g., prior treatments, genetic markers, inflammatory indices) using advanced statistical methods (e.g., machine learning approaches) could help identify significant predictors for PFS and OS. Adverse event rates are also lower than in the clinical trials. This is a concern in many registries (Dreyer et al. 2008). Underreporting is the main reason for this issue. In clinical trials, more frequent diagnostic procedures could lead to the higher adverse event rate in those studies. While this is necessary during drug development to accurately capture all safety concerns, these frequent diagnostic measures may not be necessary in real-world studies. Consequently, real-world studies, such as the present one, may be appropriate to assess the incidence of adverse events that are relevant to the actual management of therapy in routine clinical practice.

In conclusion, we could show that nab-paclitaxel is used in patients with advanced breast cancer. Furthermore, nab-paclitaxel therapy was associated with a favorable prognosis and manageable safety profile in this real-world non-interventional study.

**Table 1:** Patient and disease characteristics (efficacy population, *N* = 420)

Characteristics		mean (SD) or *N* (%)
Age (years)		60.0 (11.6)
BMI (kg/m^2^)		26.0 (5.3)
Grading at diagnosis	G1	15 (4.4)
	G2	196 (57.3)
	G3	131 (38.3)
HR status at diagnosis	HRpos	77 (20.4)
	HRneg	301 (79.6)
HER2 status at diagnosis	HER2neg	323 (94.2)
	HER2pos	20 (5.8)
Nodal status at diagnosis	pN0	112 (38.0)
	pN+	153 (51.9)
	pNX	30 (10.2)
Metastasis at diagnosis	cM0	261 (75.0)
	cM1	87 (25.0)
Metastasis pattern	Brain	33 (8.3)
	Visceral	260 (65.2)
	Bone	25 (6.3)
	Others	81 (20.3)
Karnofsky Index	< 70%	26 (6.8)
	70 − 100%	356 (93.2)
(neo)adjuvant chemotherapy	no	43 (16.7)
	yes	215 (83.3)
(neo)adjuvant endocrine therapy	no	340 (94.2)
	yes	21 (5.8)
Treatment line	first	123 (30.1)
	second	88 (21.6)
	third or more	197 (48.3)

[HR: hormone receptor: HER2: human epidermal growth factor receptor 2; pos: positive; neg: negative; SD: standard deviation; BMI: body mass index]

**Table 2:** Multivariable Cox regression model for progression-free survival and overall survival (survival analysis population, *N* = 420)

Predictor		PFS hazard ratio (95% CI)	OS hazard ratio (95% CI)
Age (year)	< 55	Reference	-^1^
	55–65	1.22 (0.80, 1.85)	-
	≥ 66	1.40 (0.95, 2.05)	-
BMI (kg/m^2^)	< 20	1.35 (0.68, 2.68)	1.30 (0.61, 2.77)
	20–25	Reference	Reference
	25–30	1.30 (0.86, 1.97)	1.06 (0.67, 1.67)
	> 30	1.36 (0.84, 2.19)	1.11 (0.66, 1.86)
Grading	G1/G2	Reference	Reference
	G3	1.07 (0.74, 1.54)	1.08 (0.71, 1.63)
HR status	HRneg	Reference	Reference
	HRpos	0.81 (0.52, 1.26)	0.72 (0.44, 1.18)
HER2 status	HER2neg	Reference	Reference
	HER2pos	1.35 (0.62, 2.92)	1.45 (0.65, 3.20)
Nodal status	pN0	Reference	Reference
	pN+	1.43 (0.98, 2.09)	1.30 (0.86, 1.98)
Metastasis pattern	Brain	1.81 (0.86, 3.79)	2.23 (1.07, 4.66)
	Visceral	Reference	Reference
	Bone	0.48 (0.21, 1.09)	0.66 (0.28, 1.56)
	Others	0.61 (0.40, 0.95)	0.47 (0.28, 0.78)
Therapy line	first	-	Reference
	second	-	1.19 (0.70, 2.03)
	third or higher	.	1.35 (0.84, 2.17)

^1^ The proportional hazards assumptions are not met for “age at diagnosis”-categories for overall survival. Age at diagnosis was used instead as a stratification variable

^2^ The proportional hazards assumptions are not met for “therapy line”-categories for progression-free survival. Therapy line was used instead as a stratification variable

*[HR: hormone receptor: HER2: human epidermal growth factor receptor 2; pos: positive; neg: negative; BMI: body mass index: PFS: progression-free survival; OS: overall survival]*

**Table 3:** Reported adverse events with a frequency over 3% (all grades) in the safety population (*N* = 432)

CTCAE Term	All Grades *N* (%)	Grade 3/4 *N* (%)
Peripheral sensory neuropathy	192 (44.4)	19 (4.4)
Malaise	130 (30.1)	6 (1.4)
Alopecia	75 (17.4)	6 (1.4)
Nausea	71 (16.4)	3 (0.7)
Diarrhea	63 (14.6)	1 (0.2)
Neutrophil count decreased	57 (13.2)	33 (7.6)
Dyspnea	54 (12.5)	1 (0.2)
Mucositis oral	43 (10)	2 (0.5)
Anemia	41 (9.5)	11 (2.5)
Rhinitis infective	38 (8.8)	0 (0)
Arthralgia	36 (8.3)	0 (0)
White blood cell decreased	36 (8.3)	15 (3.5)
Dysgeusia	31 (7.2)	0 (0)
Constipation	30 (6.9)	1 (0.2)
Edema limbs	28 (6.5)	0 (0)
Anorexia	27 (6.2)	2 (0.5)
Nail discoloration	27 (6.2)	0 (0)
Dizziness	26 (6)	1 (0.2)
Bone pain	24 (5.6)	0 (0)
Epistaxis	21 (4.9)	0 (0)
Headache	21 (4.9)	0 (0)
Myalgia	21 (4.9)	0 (0)
Vomiting	21 (4.9)	1 (0.2)
Rash maculo-papular	20 (4.6)	0 (0)
Investigations - Other, specify	18 (4.2)	3 (0.7)
Pain in extremity	18 (4.2)	0 (0)
Cough	17 (3.9)	1 (0.2)
Pain	17 (3.9)	2 (0.5)
Stomach pain	16 (3.7)	0 (0)
Lymphedema	14 (3.2)	0 (0)
Skin and subcutaneous tissue disorders - Other, specify	14 (3.2)	0 (0)
Alanine aminotransferase increased	13 (3)	0 (0)
Nail loss	13 (3)	0 (0)
Platelet count decreased	13 (3)	4 (0.9)
Urinary tract infection	13 (3)	0 (0)

[CTCAE: Common Terminology Criteria for Adverse Events]

**Table 4:** Quality of life assessment, as assessed with the FACT-B questionnaire, showing mean scores and standard deviation in efficacy population (*N* = 420 patients)

	Baseline		Month 3		Month 6	
	Response *N* (%)	Score Mean (SD)	Response *N* (%)	Score Mean (SD)	Response *N* (%)	Score Mean (SD)
FACT-B TOI	189 (43.8)	60.8 (15.8)	142 (32.9)	57.7 (16.4)	76 (17.6)	60.4 (14.7)
FACT-B total score	185 (42.8)	98.7 (21.0)	140 (32.4)	94.8 (22.0)	74 (17.1)	99.0 (21.2)
Physical well-being	193 (44.7)	19.3 (6.1)	147 (34.0)	18.4 (6.3)	76 (17.6)	18.9 (5.4)
Social well-being	188 (43.5)	22.0 (5.4)	146 (33.8)	21.3 (5.5)	75 (17.4)	21.8 (5.6)
Emotional well-being	191 (44.2)	15.6 (4.7)	145 (33.6)	15.8 (5.0)	76 (17.6)	16.6 (5.0)
Functional well-being	193 (44.7)	15.7 (6.1)	148 (34.3)	14.5 (6.0)	77 (17.8)	15.6 (5.9)
Breast Cancer Subscale	193 (44.7)	26.0 (6.5)	143 (33.1)	24.8 (6.9)	77 (17.8)	25.8 (6.5)

[SD: standard deviation]

## Electronic supplementary material

Below is the link to the electronic supplementary material.

Supplementary Material 1

## Data Availability

The data that support the findings of this study are available from the corresponding author upon reasonable request.
