# Supplementary material for: The efficacy and therapy management of nab-paclitaxel in the real-world setting for patients with advanced breast cancer – the SERAPHINA study
Source: J Cancer Res Clin Oncol. 2025 Jun 19;151(6):192. doi: 10.1007/s00432-025-06246-2 (PMC12178961; doi:10.1007/s00432-025-06246-2)
Supplement: Supplementary file 1 — Supplementary Material 1 [file 432_2025_6246_MOESM1_ESM.docx]

Supplementary material

Supplementary Table 1: List of participating study sites

| **City; Study Site Name** |
| --- |
| Aalen; Ostalbklinik; Frauenklinik |
| Amberg; Klinikum St. Marien; Praxis für Hämatologie und Internistische Onkologie |
| Aschaffenburg; Klinik; Hämato-Onkologische Schwerpunktpraxis |
| Augsburg; Klinikum; Frauenklinik |
| Bamberg; Klinikum; Sozialstiftung Bamberg |
| Bayreuth; Klinik Hohe Warte; Frauenklinik |
| Berlin; Charité; Schwerpunkt Hämatologie und Onkologie |
| Berlin; Gemeinschaftspraxis; Frauenheilkunde |
| Bonn; Clinical Cancer Center; Onkologisches Zentrum |
| Chemnitz; Poliklinik; Gynäkologie und Geburtshilfe |
| Darmstadt; Klinikum; Frauenklinik |
| Dresden; Praxis; Onkozentrum |
| Dresden; Universitätsklinikum; Frauenheilkunde und Geburtshilfe |
| Düsseldorf; Universitätsklinik; Frauenheilkunde und Geburtshilfe |
| Eggenfelden; Rottal-Inn Kliniken; Brustzentrum |
| Erlangen; Universitätsklinik; Frauenklinik |
| Essen; Universitätsklinik; Frauenklinik |
| Esslingen; Klinikum; Frauenheilkunde |
| Frankfurt; Agaplesion Markus Krankenhaus; Brustzentrum |
| Frankfurt; Onkologie Bethanien; Centrum für Hämatologie |
| Frechen; Praxis; Onkologie und Hämatologie |
| Freiburg; Universitätsklinik; Frauenheilkunde |
| Georgsmarienhütte; Franziskushospital Harderberg; Med. Versorgungszentrum |
| Goslar; Praxis; Onkologische Kooperation Harz |
| Göttingen; Praxis; Hämatologie/Onkologie |
| Hamburg-Harburg; Praxisklinik; Gynäkologische Praxisklinik |
| Hannover; Medizinische Hochschule Hannover; Frauenklinik |
| Heidelberg; Universitätsklinik; Frauenklinik/NCT |
| Homburg; Universitätsklinikum; Klinik für Frauenheilkunde |
| Jena; Universitätsklinik; Frauenklinik |
| Kaiserslautern; Schwerpunktpraxis; Hämatologie/Onkologie |
| Karlsruhe; St. Vincentius-Kliniken gAG; Geburts- und Frauenheilkunde |
| Karlsruhe; Städtisches Klinikum; Brustzentrum |
| Kassel; Klinikum; Frauenheilkunde und Geburtshilfe |
| Kiel; Universitätsklinik; Frauenheilkunde |
| Köln; Klinikum; Brustzentrum |
| Köln; Schwerpunktpraxis; Gynälologische Onkologie |
| Köln; Universitätsklinik; Frauenheilkunde und Geburtshilfe |
| Krefeld; HELIOS Kliniken; Frauenheilkunde und Geburtshilfe |
| Lahr; Klinikum; Brustzentrum |
| Landshut; Tagesklinik; Hämatologie/Onkologie/Palliativmedizin |
| Leipzig; St. Elisabeth-Krankenhaus; Brustzentrum |
| Leipzig; Universitätsklinik; Krebszentrum (UCCL) |
| Ludwigsfelde-Teltow; Evangelisches Krankenhaus; Gynäkologie |
| Mannheim; Praxisklinik am Rosengarten; Frauenarzt |
| Mannheim; Universitätsklinik; Gynäkologisches Krebszentrum |
| Minden; Mühlenkreiskliniken; Brustzentrum |
| Mönchengladbach; Evangelisches Krankenhaus; Brustzentrum Niederrhein |
| Mühlhausen; Gemeinschaftspraxis; Praxis für Frauenheilkunde |
| München; LMU Standort Innenstadt; Frauenklinik |
| München; Praxis; MVZ Onkologie |
| Münster; Universitätsklinikum; Brustzentrum |
| Nauen; Havelland Kliniken; Gynäkologie und Geburtshilfe |
| Neuruppin; Ruppiner Kliniken GmbH; Gynäkologie und Geburtshilfe |
| Nürnberg; Gemeinschaftspraxis; Frauenärzte am Stadtpark |
| Nürnberg; Klinikum Nürnberg Nord; Frauenheilkunde |
| Ostfildern-Ruit; medius Kliniken; Frauenheilkunde |
| Rodewisch; Klinikum; Frauenklinik |
| Schwerte; Marienkrankenhaus; Frauenklinik |
| Singen; Schwerpunktpraxis; Gastroenterologie |
| Soest; Kloster Paradiese; Hämatologie/Onkologie/Allgemeinmedizin |
| Speyer; Schwerpunktpraxis; Onkologische Schwerpunktpraxis |
| Stendal; Johanniter-Krankenhaus; Brustzentrum |
| Stralsund; Kompetenzzentrum; Frauenheilkunde und Geburtshilfe |
| Stuttgart; Facharztzentrum; Brustzentrum Femininum |
| Torgau; Kreiskrankenhaus; Brustzentrum |
| Troisdorf; Schwerpunktpraxis; Hämatologie/Onkologie |
| Tübingen; Universitätsklinik; Frauenklinik |
| Weiden; Medizinisches Versorgungszentrum; Onkologie |
| Weinheim; GRN-Klinik; Gynäkologie |
| Westerstede; Praxis; Onkologie |
| Wetzlar; Klinikum; Frauenklinik |
| Witten; Gemeinschaftspraxis; Innere Medizin |
| Wolfenbüttel; Gemeinschaftspraxis; Gynäkologie/Onkologie |
| Würselen; Praxis; Hämatologie/Onkologie |
| Würzburg; Praxis; Frauenheilkunde und Geburtshilfe |

Supplementary Table 2: Nab-paclitaxel therapy schedules and therapy management in the safety population (N=432)

| **Characteristic** |  | **N (%) or mean (SD)** |
| --- | --- | --- |
| Initially planned therapy regimen | Weekly | 167 (38.8) |
|  | Every two weeks | 9 (2.1) |
|  | Cycle of three weeks | 105 (24.4) |
|  | Cycle of four weeks | 149 (34.7) |
| Has a dose reduction ever occured? | Yes | 158 (36.6) |
|  | No | 274 (63.4) |
| Has the dosing interval ever been adjusted? | Yes | 112 (25.9) |
|  | No | 320 (74.1) |
| Reason for end of therapy | Regular end of study | 24 (5.6) |
|  | Patient dead | 284 (65.7) |
|  | Withdrawel of consent | 15 (3.5) |
|  | Patient is lost to follow up | 33 (7.6) |
|  | Missing information | 76 (17.6) |
| Nab-paclitaxel therapy | Duration (weeks) | 20.87 (45.52) |
|  | Number of doses | 13.12 (9.00) |
|  | Cumulative dose (mg) | 2555.71 (1830.37) |
|  | Relative dose intensity (%) | 0.91 (0.44) |

*[SD: standard deviation]*

Supplementary Table 3: Median survival times and survival rates (efficacy population N=420)

| **Outcome** | **Patients** | **Events** | **Median  survival time (95% CI)** | **6-month  survival rate (95% CI)** | **12-month  survival rate (95% CI)** | **24-month survival time (95% CI)** |
| --- | --- | --- | --- | --- | --- | --- |
| PFS | 420 | 359 | 6.0 (5.6, 6.9) | 0.50 (0.46, 0.56) | 0.24 (0.20, 0.28) | 0.11 (0.08, 0.15) |
| OS | 420 | 279 | 15.3 (12.5, 17.5) | 0.81 (0.77, 0.85) | 0.57 (0.52, 0.62) | 0.35 (0.31, 0.41) |

*[CI: confidence interval; PFS; progression-free survival; OS: overall survival]*

Supplementary Table 4: Progression-free survival according to patient subgroups (efficacy population, N=420).

| **Characteristics** |  | **N** | **Events** | **Median  survival time (months)**  **(95% CI)** | **6-month  survival rate (95% CI)** | **12-month survival rate (95% CI)** | **24-month survival time (95% CI)** |
| --- | --- | --- | --- | --- | --- | --- | --- |
| Age (years) | < 55 | 142 | 123 | 6.0 (5.3, 9.0) | 0.50 (0.42, 0.59) | 0.30 (0.24, 0.39) | 0.13 (0.08, 0.20) |
|  | 55-65 | 131 | 115 | 5.6 (4.8, 6.8) | 0.47 (0.39, 0.57) | 0.19 (0.13, 0.27) | 0.10 (0.06, 0.17) |
|  | ≥ 66 | 146 | 120 | 6.4 (5.6, 7.9) | 0.53 (0.46, 0.62) | 0.21 (0.15, 0.29) | 0.11 (0.07, 0.18) |
| BMI (kg/m^2^) | < 20 | 37 | 31 | 8.2 (4.0, 10.1) | 0.56 (0.41, 0.74) | 0.20 (0.11, 0.39) | 0.14 (0.06, 0.32) |
|  | 20-25 | 179 | 153 | 6.7 (5.7, 8.2) | 0.55 (0.48, 0.63) | 0.25 (0.19, 0.33) | 0.11 (0.07, 0.17) |
|  | 25-30 | 112 | 95 | 5.7 (5.3, 6.9) | 0.46 (0.38, 0.57) | 0.21 (0.14, 0.31) | 0.11 (0.06, 0.19) |
|  | > 30 | 86 | 74 | 5.6 (4.5, 7.1) | 0.44 (0.34, 0.56) | 0.27 (0.19, 0.39) | 0.13 (0.07, 0.23) |
| Grading | G1/2 | 211 | 183 | 5.9 (5.3, 6.6) | 0.47 (0.41, 0.55) | 0.22 (0.17, 0.29) | 0.09 (0.06, 0.15) |
|  | G3 | 131 | 117 | 6.1 (4.6, 8.2) | 0.50 (0.42, 0.60) | 0.20 (0.14, 0.29) | 0.09 (0.05, 0.16) |
| HR status | HRneg | 77 | 67 | 5.3 (4.1, 6.9) | 0.44 (0.34, 0.57) | 0.25 (0.17, 0.38) | 0.13 (0.07, 0.24) |
|  | HRpos | 301 | 261 | 6.2 (5.7, 7.0) | 0.52 (0.46, 0.58) | 0.22 (0.18, 0.27) | 0.09 (0.06, 0.13) |
| HER2 status | HER2neg | 323 | 281 | 6.0 (5.6, 6.9) | 0.50 (0.45, 0.56) | 0.23 (0.18, 0.28) | 0.10 (0.07, 0.14) |
|  | HER2pos | 20 | 17 | 5.5 (2.1, 22.3) | 0.49 (0.31, 0.77) | 0.30 (0.15, 0.61) | 0.18 (0.07, 0.50) |
| Nodal status | pN0 | 112 | 96 | 6.0 (5.1, 7.6) | 0.49 (0.40, 0.59) | 0.26 (0.19, 0.36) | 0.12 (0.07, 0.20) |
|  | pN+ | 153 | 141 | 5.7 (4.6, 6.4) | 0.45 (0.38, 0.54) | 0.17 (0.12, 0.24) | 0.06 (0.03, 0.11) |
|  | pNX | 30 | 28 | 7.1 (4.8, 10.3) | 0.55 (0.40, 0.77) | 0.17 (0.08, 0.38) | 0.04 (0.01, 0.28) |
| Metastasis at diagnosis | cM0 | 261 | 226 | 5.7 (5.3, 6.7) | 0.48 (0.42, 0.54) | 0.20 (0.15, 0.26) | 0.10 (0.07, 0.15) |
|  | cM1 | 87 | 71 | 6.0 (4.6, 9.4) | 0.50 (0.40, 0.62) | 0.29 (0.20, 0.41) | 0.15 (0.09, 0.27) |
| Metastasis pattern | brain | 33 | 32 | 3.0 (2.2, 4.3) | 0.15 (0.07, 0.35) | 0.06 (0.02, 0.24) | 0.03 (0.00, 0.21) |
|  | visceral | 260 | 223 | 6.0 (5.5, 7.0) | 0.50 (0.44, 0.57) | 0.20 (0.16, 0.26) | 0.09 (0.06, 0.14) |
|  | bone | 25 | 19 | 8.2 (4.7, 20.9) | 0.61 (0.44, 0.85) | 0.42 (0.26, 0.69) | 0.21 (0.09, 0.49) |
|  | others | 81 | 70 | 8.0 (5.7, 10.9) | 0.59 (0.49, 0.71) | 0.33 (0.24, 0.45) | 0.13 (0.07, 0.24) |
| Karnofsky Index | < 70% | 26 | 23 | 3.3 (2.0, 9.0) | 0.31 (0.17, 0.56) | 0.09 (0.03, 0.34) | 0.05 (0.01, 0.31) |
|  | 70% - 100% | 356 | 301 | 6.4 (5.9, 7.2) | 0.53 (0.48, 0.59) | 0.26 (0.22, 0.31) | 0.13 (0.09, 0.17) |
| (neo)adjuvant chemotherapy | no | 43 | 32 | 5.4 (3.3, 10.6) | 0.44 (0.31, 0.63) | 0.19 (0.10, 0.37) | 0.16 (0.08, 0.34) |
|  | yes | 215 | 191 | 5.9 (5.3, 6.8) | 0.48 (0.42, 0.56) | 0.20 (0.15, 0.27) | 0.10 (0.06, 0.15) |
| (neo)adjuvant endocrine therapy | no | 340 | 292 | 6.0 (5.4, 6.9) | 0.50 (0.44, 0.55) | 0.23 (0.19, 0.28) | 0.10 (0.07, 0.15) |
|  | yes | 21 | 17 | 8.0 (2.4, 26.4) | 0.56 (0.38, 0.83) | 0.39 (0.22, 0.69) | 0.21 (0.08, 0.53) |
| Therapy line | first | 123 | 88 | 9.8 (7.9, 12.6) | 0.64 (0.56, 0.73) | 0.44 (0.36, 0.55) | 0.26 (0.19, 0.36) |
|  | second | 88 | 79 | 6.3 (5.4, 7.2) | 0.53 (0.43, 0.64) | 0.19 (0.12, 0.30) | 0.06 (0.02, 0.14) |
|  | third or more | 197 | 181 | 5.3 (3.9, 6.0) | 0.42 (0.36, 0.50) | 0.13 (0.09, 0.18) | 0.05 (0.03, 0.10) |

*[BMI: body mass index; CI: confidence intervall; HR: hormone receptor; HER2: human epidermal growth factor receptor 2; pos: positive; neg: negative]*

Supplementary Table 5: Overall survival according to patient subgroups (efficacy population, N=420).

| **Characteristics** |  | **N** | **Events** | **Median  survival time (months) (95% CI)** | **6-month  survival rate (95% CI)** | **12-month survival rate (95% CI)** | **24-month survival time (95% CI)** |
| --- | --- | --- | --- | --- | --- | --- | --- |
| Age (years) | < 55 | 142 | 99 | 17.1 (12.5, 20.1) | 0.84 (0.78, 0.90) | 0.60 (0.52, 0.69) | 0.34 (0.26, 0.43) |
|  | 55-65 | 131 | 82 | 14.0 (10.1, 24.9) | 0.78 (0.71, 0.86) | 0.54 (0.45, 0.64) | 0.41 (0.33, 0.51) |
|  | ≥ 66 | 146 | 98 | 12.8 (10.8, 17.4) | 0.80 (0.73, 0.87) | 0.55 (0.47, 0.64) | 0.31 (0.23, 0.41) |
| BMI (kg/m^2^) | < 20 | 37 | 18 | 17.5 (10.7, NA) | 0.81 (0.69, 0.95) | 0.62 (0.48, 0.81) | 0.44 (0.30, 0.66) |
|  | 20-25 | 179 | 121 | 12.7 (10.7, 17.5) | 0.79 (0.73, 0.86) | 0.53 (0.46, 0.62) | 0.35 (0.28, 0.44) |
|  | 25-30 | 112 | 80 | 17.3 (12.3, 20.6) | 0.80 (0.72, 0.88) | 0.59 (0.51, 0.69) | 0.33 (0.25, 0.44) |
|  | > 30 | 86 | 55 | 16.6 (11.2, 23.3) | 0.84 (0.76, 0.92) | 0.57 (0.47, 0.70) | 0.35 (0.26, 0.49) |
| Grading | G1/2 | 211 | 140 | 14.2 (11.3, 17.5) | 0.77 (0.71, 0.83) | 0.55 (0.48, 0.63) | 0.34 (0.27, 0.42) |
|  | G3 | 131 | 94 | 14.3 (10.7, 18.4) | 0.86 (0.80, 0.92) | 0.55 (0.47, 0.65) | 0.29 (0.22, 0.39) |
| HR status | HRneg | 77 | 50 | 12.5 (10.2, 20.6) | 0.78 (0.69, 0.88) | 0.53 (0.42, 0.66) | 0.33 (0.23, 0.47) |
|  | HRpos | 301 | 203 | 16.0 (12.6, 18.3) | 0.83 (0.78, 0.87) | 0.58 (0.52, 0.64) | 0.36 (0.30, 0.42) |
| HER2 status | HER2neg | 323 | 218 | 16.2 (12.6, 18.2) | 0.82 (0.78, 0.86) | 0.58 (0.53, 0.64) | 0.36 (0.30, 0.42) |
|  | HER2pos | 20 | 13 | 10.2 (6.9, NA) | 0.74 (0.57, 0.96) | 0.43 (0.25, 0.75) | 0.30 (0.14, 0.63) |
| Nodal status | pN0 | 112 | 77 | 13.5 (10.2, 18.5) | 0.79 (0.72, 0.88) | 0.55 (0.46, 0.65) | 0.32 (0.23, 0.42) |
|  | pN+ | 153 | 105 | 13.2 (10.7, 18.2) | 0.81 (0.75, 0.88) | 0.55 (0.47, 0.64) | 0.33 (0.26, 0.42) |
|  | pNX | 30 | 22 | 14.3 (10.0, 29.7) | 0.79 (0.66, 0.96) | 0.55 (0.40, 0.77) | 0.37 (0.23, 0.60) |
| Metastasis at diagnosis | cM0 | 261 | 175 | 13.5 (10.6, 17.5) | 0.80 (0.75, 0.85) | 0.53 (0.47, 0.60) | 0.33 (0.28, 0.40) |
|  | cM1 | 87 | 60 | 17.3 (12.5, 22.9) | 0.81 (0.73, 0.90) | 0.63 (0.53, 0.74) | 0.36 (0.26, 0.49) |
| Metastasis pattern | brain | 33 | 30 | 7.6 (5.8, 13.2) | 0.62 (0.47, 0.81) | 0.29 (0.17, 0.51) | 0.11 (0.04, 0.31) |
|  | visceral | 260 | 184 | 13.1 (10.9, 17.1) | 0.80 (0.75, 0.85) | 0.54 (0.48, 0.61) | 0.32 (0.26, 0.39) |
|  | bone | 25 | 11 | 26.2 (14.3, NA) | 0.82 (0.67, 1.00) | 0.77 (0.61, 0.97) | 0.52 (0.34, 0.79) |
|  | others | 81 | 47 | 18.5 (14.2, 32.4) | 0.86 (0.79, 0.94) | 0.62 (0.52, 0.74) | 0.45 (0.35, 0.58) |
| Karnofsky Index | < 70% | 26 | 19 | 6.9 (3.1, 17.5) | 0.53 (0.36, 0.77) | 0.23 (0.10, 0.51) | 0.15 (0.05, 0.47) |
|  | 70% - 100% | 356 | 234 | 16.6 (13.2, 19.0) | 0.83 (0.79, 0.87) | 0.59 (0.54, 0.65) | 0.38 (0.33, 0.44) |
| (neo)adjuvant chemotherapy | no | 43 | 24 | 16.2 (8.9, NA) | 0.74 (0.61, 0.89) | 0.54 (0.40, 0.73) | 0.33 (0.20, 0.54) |
|  | yes | 215 | 148 | 13.5 (10.7, 17.8) | 0.81 (0.76, 0.87) | 0.54 (0.47, 0.61) | 0.34 (0.27, 0.41) |
| (neo)adjuvant endocrine therapy | no | 340 | 230 | 15.5 (12.5, 17.8) | 0.82 (0.77, 0.86) | 0.57 (0.52, 0.63) | 0.35 (0.30, 0.41) |
|  | yes | 21 | 14 | 19.0 (10.1, NA) | 0.79 (0.63, 1.00) | 0.57 (0.38, 0.85) | 0.39 (0.22, 0.70) |
| Therapy line | first | 123 | 64 | 25.7 (20.5, 37.0) | 0.85 (0.79, 0.92) | 0.67 (0.59, 0.76) | 0.55 (0.46, 0.65) |
|  | second | 88 | 60 | 15.8 (11.5, 20.1) | 0.82 (0.74, 0.91) | 0.59 (0.49, 0.71) | 0.34 (0.25, 0.47) |
|  | third or more | 197 | 146 | 11.4 (9.7, 14.2) | 0.77 (0.71, 0.83) | 0.49 (0.42, 0.57) | 0.25 (0.19, 0.33) |

*[BMI: body mass index; CI: confidence intervall; HR: hormone receptor; HER2: human epidermal growth factor receptor 2; pos: positive; neg: negative]*

Supplementary Figure 1: The study flow chart.


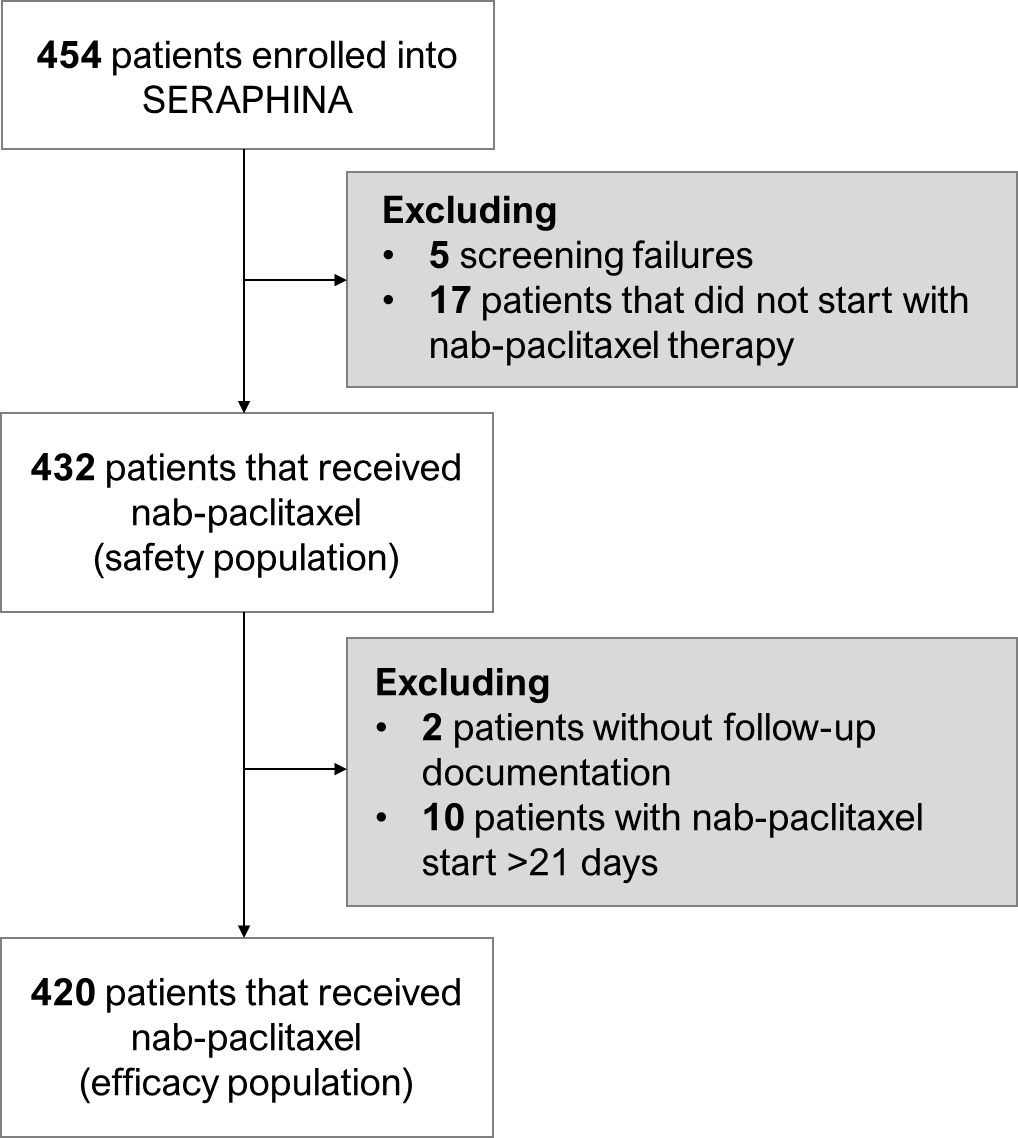


Supplementary Figure 2: Progression-free survival (a) and overall survival (b) according to age

| **a** | 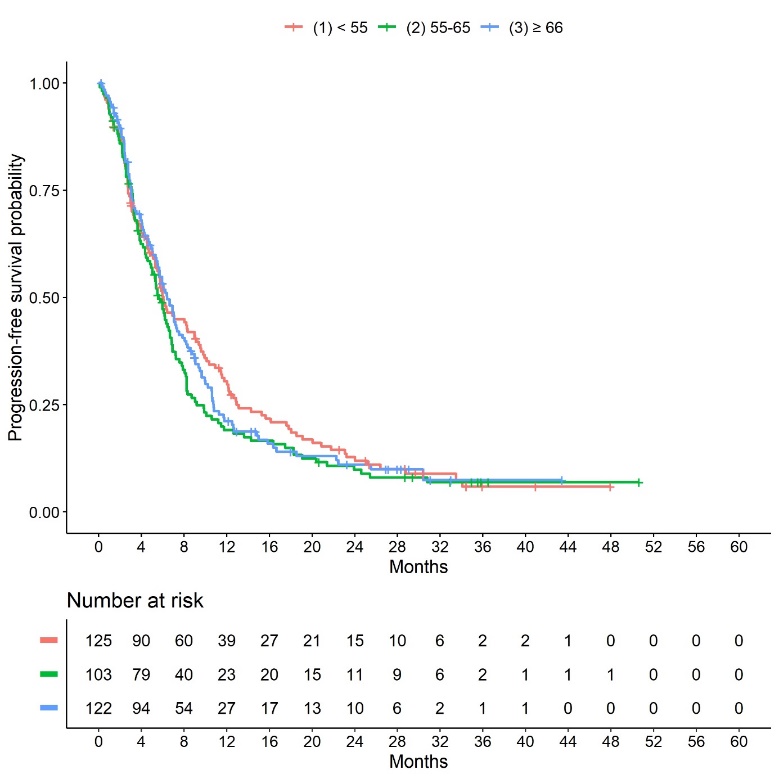 |
| --- | --- |
| **b** | 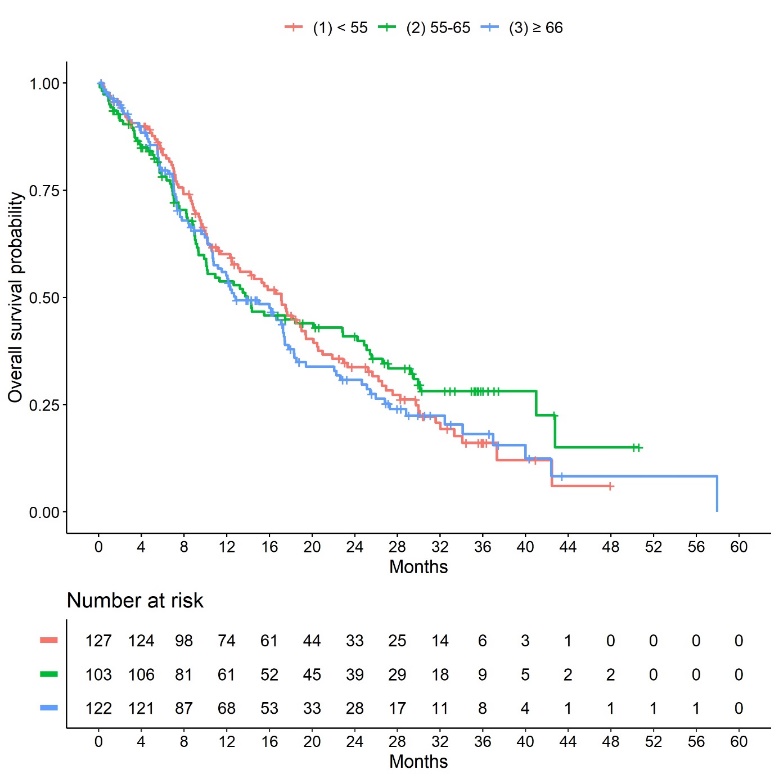 |

Supplementary Figure 3: Progression-free survival (a) and overall survival (b) according to metastasis pattern

| **a** | 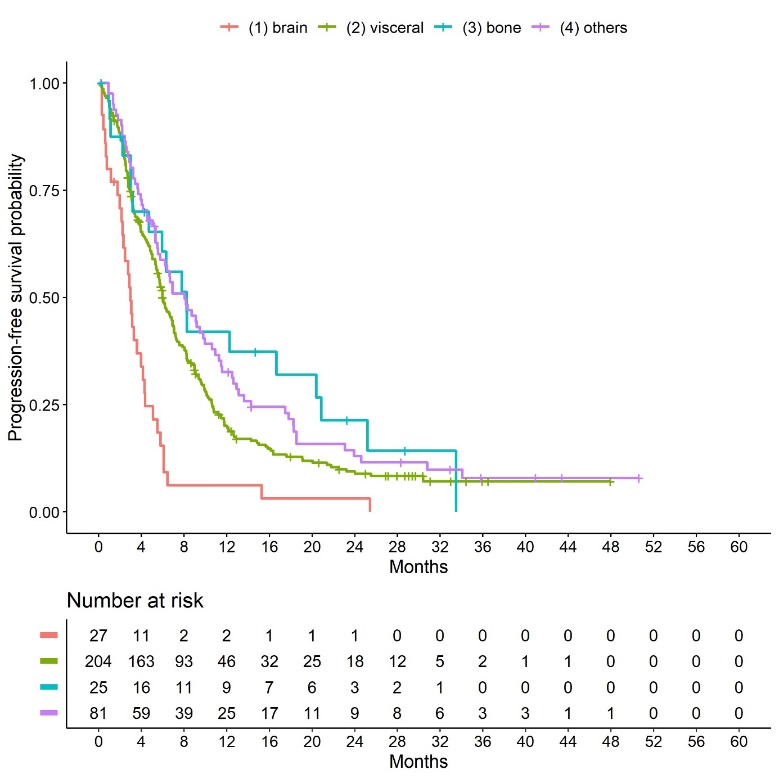 |
| --- | --- |
| **b** | 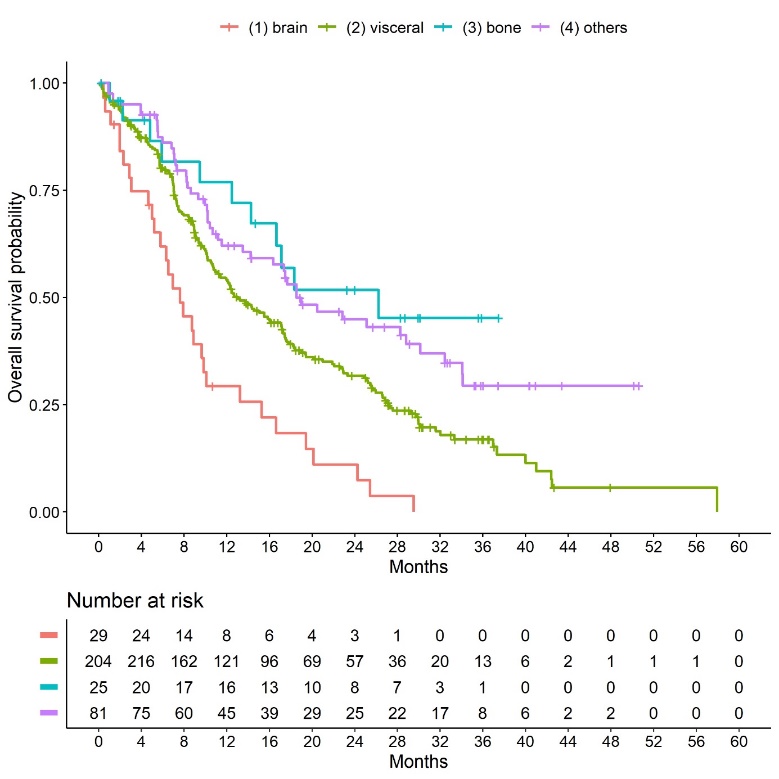 |

Supplementary Figure 4: Progression-free survival (a) and overall survival (b) according to Karnofsky Index

| **a** | 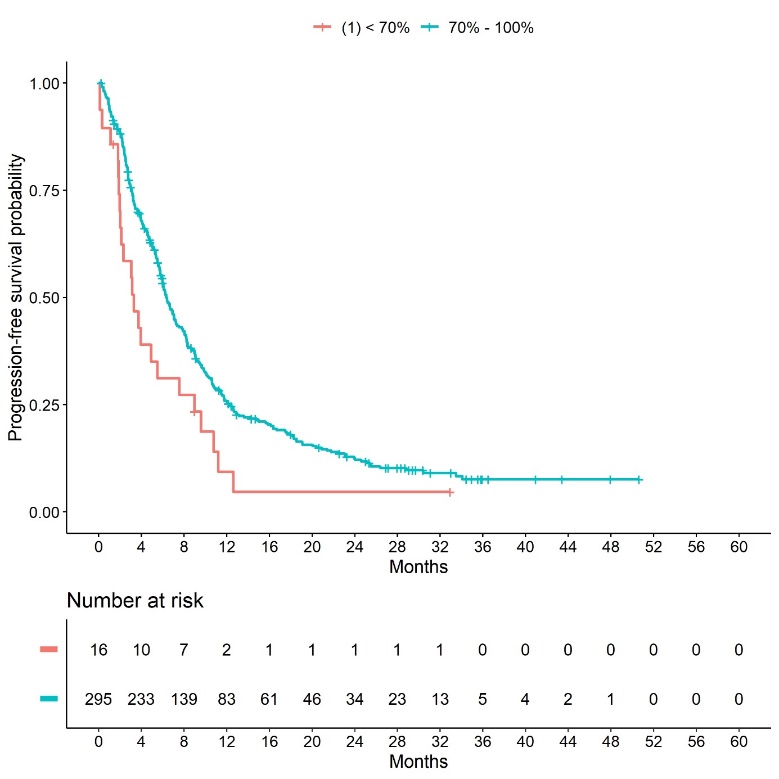 |
| --- | --- |
| **b** | 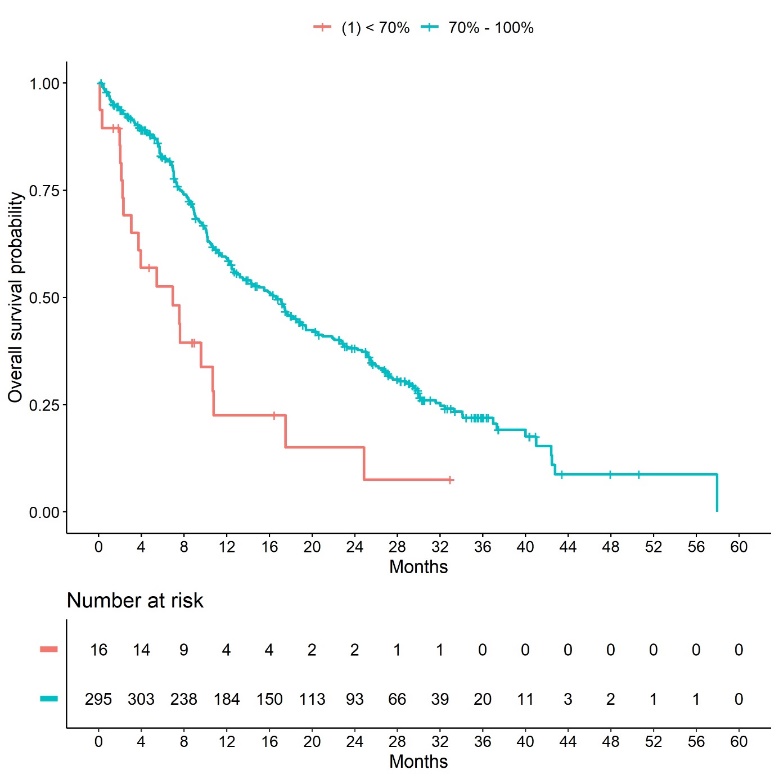 |

Supplementary Figure 5: Progression-free survival (a) and overall survival (b) according to therapy line

| **a** | 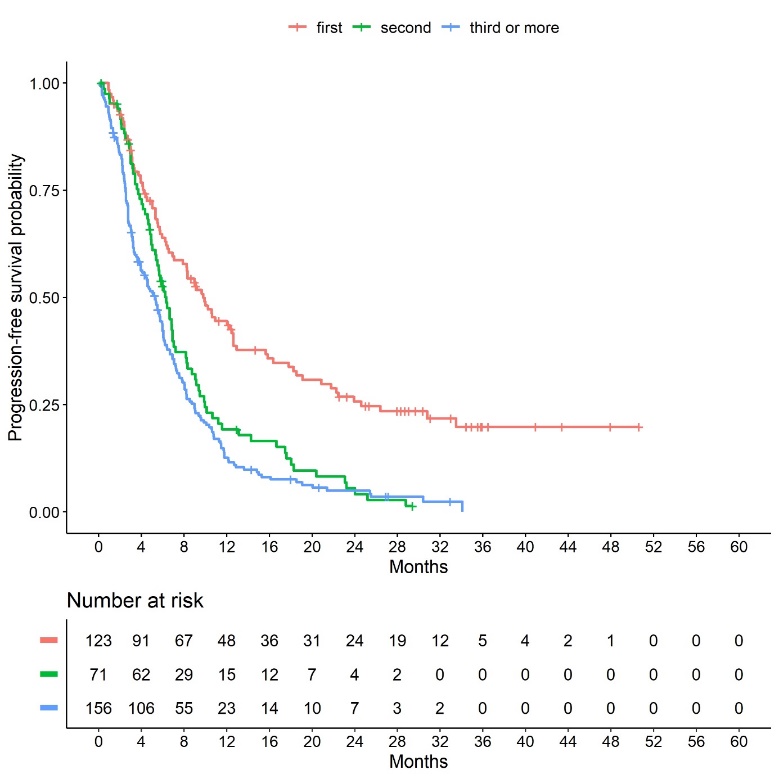 |
| --- | --- |
| **b** | 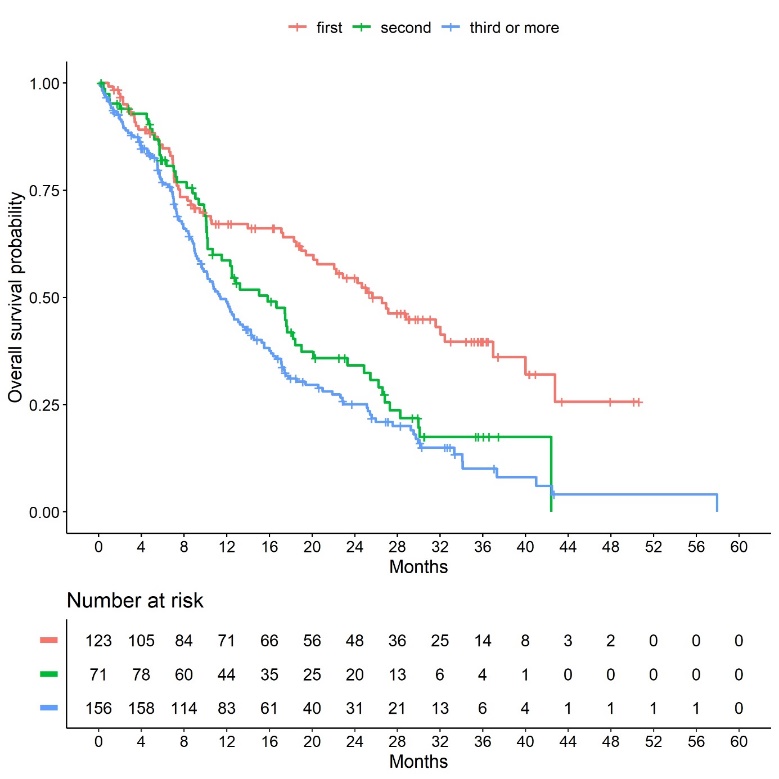 |
